# Molecular Diagnosis of *Felis catus* Gammaherpesvirus 1 (FcaGHV1) Infection in Cats of Known Retrovirus Status with and without Lymphoma

**DOI:** 10.3390/v10030128

**Published:** 2018-03-14

**Authors:** Alicia J. McLuckie, Vanessa R. Barrs, Scott Lindsay, Mahdis Aghazadeh, Cheryl Sangster, Julia A. Beatty

**Affiliations:** 1 Sydney School of Veterinary Science, Faculty of Science, The University of Sydney, Sydney, NSW 2006, Australia; amcl8135@uni.sydney.edu.au (A.J.M.); vanessa.barrs@sydney.edu.au (V.R.B.); mahdis.aghazadeh@sydney.edu.au (M.A.); 2 School of Animal and Veterinary Sciences, Faculty of Science, The University of Adelaide, Adelaide, SA 5005, Australia; scott.lindsay@adelaide.edu.au; 3 Veterinary Pathology Diagnostic Services, Sydney School of Veterinary Science, Faculty of Science, The University of Sydney, Sydney, NSW 2006, Australia; cheryl.sangster@sydney.edu.au

**Keywords:** gammaherpesvirus, cat, feline, lymphoma, cancer, survival, domestic cats, disease association, retrovirus, FcaGHV1, FeLV, FIV, pathogenesis

## Abstract

The pathogenicity of *Felis catus* gammaherpesvirus 1 (FcaGHV1), a common infection of domestic cats, is unknown. To explore an association between FcaGHV1 detection and feline lymphoma, a retrospective, cross-sectional, disease-association study was conducted. The infection status of all cats for feline immunodeficiency virus and feline leukaemia virus was determined. Neither a molecular diagnosis of FcaGHV1 nor whole-blood FcaGHV1 load was related to outcome in 122 lymphoma cases compared with 71 controls matched for age and sex. Molecular analysis of lymphoma-derived DNA paired with autologous uninvolved tissue did not suggest restriction of FcaGHV1 DNA to tumour tissue. FcaGHV1 DNA detection was associated with significantly shorter survival in lymphoma cases, an observation that could not be adequately explained by treatment differences. In addition, regressive feline leukaemia virus infection was identified as a risk factor for lymphoma. A history of fighting or roaming was identified as a novel epidemiological risk factor for FcaGHV1 detection, lending support to intercat aggression as a potential route of transmission. Studies investigating the cellular location and expression of FcaGHV1 are indicated to assist in ruling out a lymphomagenic role for this virus. Prospective investigation of FcaGHV1 DNA detection as a prognostic marker in feline lymphoma is warranted.

## 1. Introduction

*Gammaherpesvirinae*, a subfamily in *Herpesviridae*, co-evolved with a diverse range of mammals [1]. Asymptomatic infection is the norm, with host immunity expected to eliminate virus from all but a few persistently infected cells [2]. However, when viral containment is compromised, gammaherpesviruses can cause cancers and other diseases that negatively impact animal and human health, particularly in the developing world [3]. For example, the two human gammaherpesviruses, Epstein-Barr virus (EBV; Human herpesvirus 4) and Kaposi’s sarcoma-associated herpesvirus (KSHV; Human herpesvirus 8), together cause over 160,000 new cancer cases annually [4,5].

*Felis catus* gammaherpesvirus 1 (FcaGHV1) was discovered in 2014 and is estimated to infect at least one quarter of the world’s 600 million domestic cats [6,7,8,9,10,11]. Virus transmission during aggressive encounters is suggested by common epidemiological factors for fighting and detection of FcaGHV1 DNA in blood (DNAemia), including male sex and coinfections with feline immunodeficiency virus (FIV) and haemoplasmas [9,12,13]. Like other gammaherpesviruses, FcaGHV1 is lymphotropic and can be detected in circulating B-cells and CD4+ and CD8+ T-cells [14]. To date, FcaGHV1 tissue tropism has been investigated in post-mortem samples from three FIV-infected cats where the virus was demonstrated by qPCR in a broad range of tissues [7]. Gammherpesviruses are typically host-specific. The host range of FcaGHV1 has not been extensively investigated, but no FcaGHV1-positive PCR results were detected among whole blood samples from bobcats (*Lynx rufus*) or pumas (*Puma concolor*) [6].

Defining the oncogenic potential of FcaGHV1 is important for the welfare of domestic cats, where lymphoma is a major contributor to morbidity and mortality [15]. In the 1970s, 70% of feline lymphomas were caused by feline leukaemia virus (FeLV) infection, a directly oncogenic gammaretrovirus [16,17]. Vaccination and management procedures to prevent FeLV infection have resulted in a dramatic fall in FeLV-associated lymphoma to less than 15% of all lymphomas in cats [17,18]. Despite this progress, lymphoma remains the most common malignancy of domestic cats and there is evidence that the incidence of lymphoma may be increasing, indicating the need to explore other aetiologies [19].

Understanding the oncogenic potential of FcaGHV1 presents specific challenges. In other species, gammaherpesvirus-associated oncogenesis is a sporadic outcome of chronic infections. Additional factors, such as immune dysfunction or coinfections, are often required for expression of the full malignant phenotype [2]. Determining causality will rely on analysing accumulating evidence from multiple investigations [20]. Comparative data support the investigation of a role for FcaGHV1 infection in lymphomagenesis, particularly in cases where there is co-infection with the lentivirus FIV. Similar to human immunodeficiency virus (HIV) infection in humans, FIV-infected cats develop immune dysfunction and are at increased risk of developing lymphoma [21,22]. Of relevance here is that, depending on subtype, 30 to 95% of HIV-associated lymphomas are caused by gammaherpesviruses (EBV and/or KSHV), prompting consideration that FcaGHV1 could be a copathogen in FIV-associated lymphoma [23]. Certainly, FIV infection is a consistent risk factor for FcaGHV1 DNA detection in whole blood (DNAemia) and the FcaGHV1 load in whole blood is approximately 5 times higher in FIV-infected cats than in FIV-uninfected cats [7,8,10]. Epidemiologic data lend support to a pathogenic role for FcaGHV1; FcaGHV1 infected cats were 2.8 times more likely to be classified as sick than healthy on physical examination by a veterinarian blinded to the cat’s infection status in one study [7]. Further, FcaGHV1 is panlymphotropic, consistent with a possible role for the virus in lymphomagenesis [14].

Information derived from disease-association studies in cats of known FeLV and FIV infection status is a logical next step to inform causality. The aims of this study were to compare: (a) the prevalence of FcaGHV1 DNA detection in cats with and without lymphoma; (b) the FcaGHV1 DNA load in whole blood from cats with and without lymphoma; and (c) the frequency of FcaGHV1 DNA detection in lymphoma tissues with that in autologous uninvolved tissues and lymphoid tissues from cats without lymphoma. Our data set also allowed us to investigate the relationship between FcaGHV1 DNAemia and survival time in lymphoma cases, novel epidemiological risk factors for FcaGHV1 DNAemia and regressive FeLV infection as a risk factor for lymphoma.

## 2. Materials and Methods

### 2.1. Ethical Approval

This study was approved by the University of Sydney Animal Ethics Committee (identification codes 2014/626 and 2015/821, approved on 9 July 2014 and 19 June 2015 respectively).

### 2.2. Samples

Feline cases diagnosed with lymphoma at the University of Sydney between January 2006 and July 2017 were identified from veterinary medical and pathology records databases. Data were retrieved for cases that had a clinically aggressive disease course and either: (a) a histopathological diagnosis of high-grade lymphoma (≥4 mitoses/high powered field (hpf; averaged over 10 fields); [24]) or intermediate-grade lymphoma (>2, <4 mitoses/hpf; [24]); or (b) a histopathological diagnosis of small- to medium-cell lymphoma or low-grade lymphoma (≤2 mitoses/hpf; [24]), but only if multicentric or multiorgan involvement, or invasion into adjacent tissue planes was identified; or (c) there was a diagnosis by fine needle aspirate cytology of medium-cell or large-cell lymphoma. Cases were excluded if no remaining formalin-fixed paraffin embedded (FFPE) biopsy tissue and/or whole blood stored at −80 °C were available for study. In total, 122 lymphoma cases were available for study, comprising 93 diagnosed on histopathology (tissues available: residual biopsy and blood *n* = 44, residual biopsy only *n* = 29, and blood only *n* = 20) and 29 cases diagnosed on cytology (whole blood available).

Control samples were: (a) paired autologous uninvolved FFPE tissue from cases diagnosed on histopathology (*n* = 33); (b) FFPE lymph node from cases with a diagnosis other than lymphoma (*n* = 31); and (c) whole blood samples from cases from the same region with a diagnosis other than lymphoma, some of which have been reported previously (*n* = 86; [25]). Data on age, sex and breed were recorded for all cases and controls, and retrovirus status was recorded, where available. Regarding environment, cats were assigned to one of two categories; indoor, where confinement indoors for the cat’s lifetime was confirmed, or outdoor, where outdoor access was confirmed or could not be excluded. Similarly, the cat’s history of fighting was recorded as either observed or not observed, the latter including unknown history. For lymphoma cases, treatment categories were recorded as: no treatment, prednisolone alone, multi-agent chemotherapy, or unknown. Survival times from diagnosis to death (or euthanasia) or loss to follow-up were calculated.

### 2.3. Immunophenotyping

Immunohistochemical staining was performed using the following antibodies: CD3 (Dako, Australia Pty Ltd., North Sydney, NSW 2060, Australia, A0452, 1:400); CD79b (AbD Serotec, Abacus ALS, Meadowbrook, Qld 4131, Australia, MCA2209, 1:5000); and PAX5 (Leica Novocastra, Sigma-Aldrich, Castle Hill, NSW 1765, Australia, 1:100), as reported previously [26,27]. Briefly, 4 µm sections underwent endogenous peroxidase blocking for 15 min followed by heat-induced epitope retrieval for five minutes (Dako, S1699, S2367) and then incubation with the primary antibody at room temperature for 60 min. Binding was detected using a commercial peroxidase system (Dako K5007), and slides were counterstained with Whitlock’s hematoxylin. Positive and negative tissue controls were included in each run. All slides were reviewed by a veterinary pathologist. 

### 2.4. Virus DNA Detection

#### 2.4.1. DNA Extraction

DNA was extracted from 8 µm scrolls of FFPE tissue, cut using standard techniques to avoid contamination. QiaAMP DNA Micro Kit (Qiagen Pty Ltd., Chadstone, VIC 3148, Australia) was used for small tissue sections (≤0.5 cm^2^), or DNeasy^®^ Blood and Tissue Kit (Qiagen Pty Ltd., Chadstone, VIC 3148, Australia) for tissue sections >0.5 cm^2^, as well as for whole blood samples using 100 µL. 

#### 2.4.2. FcaGHV1 PCR

Conventional PCR (cPCR) was designed to amplify a 164 bp fragment of the FcaGHV1 *glycoprotein B* (*gB*) gene from FFPE tissue-derived DNA. Primers were designed using Oligo 7 (Molecular Biology Insights); GH-3F (forward) 5′-TGACATGTAACGCAGTCTATG-3′ and GH-3R (reverse) 5′-TCTGTGCATGATTCGTTCCAT-3′. The assay was optimised using samples confirmed to amplify the target by sequencing. Each reaction contained 300–700 ng of template DNA, 1 µM of forward and reverse primers, 1.25 units of MyTaq^TM^ HS DNA Polymerase (Bioline (Aust) Pty Ltd., Alexandria, NSW 1435, Australia) and 5 µL MyTaq^TM^ Reaction Buffer Red (Bioline (Aust) Pty Ltd., Alexandria, NSW 1435, Australia) in a total volume of 25 µL. Cycling conditions were: initial denaturation at 95 °C for one minute, followed by 40 cycles of denaturation at 95 °C for 15 s, annealing at 49.7 °C for 15 s, and extension at 72 °C for 10 s, then a final extension at 72 °C for four minutes. The lower limit of detection of the assay was determined to be 1500 viral DNA copies per reaction by titrating FcaGHV1 positive FFPE-derived DNA, where the FcaGHV1 viral load had been determined by real-time quantitative PCR (qPCR) performed on DNA derived from frozen tissue [6]. 

A qPCR targeting the FcaGHV1 *gB* gene [6] was used to detect and quantitate FcaGHV1 DNA from whole blood-derived DNA. All qPCR samples were tested in triplicate. Positive samples contained three or more DNA copies per reaction. Amplification efficiency ranged from 95 to 105% and *R*^2^ > 0.99. The lower limit of detection of the qPCR is three viral DNA copies per reaction [6], the lowest viral load detected in a clinical sample using this qPCR is 88 copies per 10^6^ host cells t [7].

#### 2.4.3. FIV and FeLV cPCR

All samples were tested for FeLV and FIV because these viruses are known risk factors for lymphoma. FIV provirus was detected using primers amplifying a 271 bp fragment of the *gag* gene (specifically p15) [28] with conditions as above, apart from an annealing temperature of 47 °C. FFPE tissue-derived DNA from an FIV-infected cat was used as a positive control. For FeLV provirus detection, primers amplifying a 120 bp fragment of U3 [29] were used with the same conditions, except the annealing temperature was 61 °C. FFPE tissue-derived DNA from a cat with progressive FeLV infection was used as a positive control. 

#### 2.4.4. cPCR Product Detection and Confirmation

No-template (molecular-grade water) and positive controls were included in all cPCR assays. Products were separated on 1.5% agarose gel containing SYBR^TM^ Safe DNA Gel Stain (Invitrogen, Carlsbad, CA, USA) in TBE buffer and were visualised under UV light (Bio-Rad Gel Doc XR System, Bio-Rad Laboratories Pty Ltd., Gladesville, NSW 2111, Australia). Positive samples were identified by a band migrating at the expected product size. The identity of PCR products from positive controls, a random selection of samples and any sample giving a band at the expected size in the FeLV cPCR were confirmed using Sanger sequencing (Macrogen Inc., Geumcheon-gu, Seoul 08511, South Korea). Any DNA sample that gave a negative result on all viral PCRs was tested for feline glyceraldehyde-3-phosphate dehydrogenase (GAPDH) by cPCR to confirm the presence of amplifiable genomic DNA and the absence of PCR inhibitors [14]. Samples that were negative for GAPDH were excluded from the analysis (lymphoma FFPE samples, *n* = 6; autologous FFPE controls, *n* = 3; lymphoma blood samples, *n* = 1; control lymph node FFPE samples, *n* = 3; total *n* = 13).

### 2.5. Individual Infection Status

The FcaGHV1, FIV and FeLV infection status of each individual was described based on the results of testing tissues alone, or in combination with historical data. A molecular diagnosis of FcaGHV1-infection was made if FcaGHV1 DNA was detected in any sample, otherwise the case was reported as FcaGHV1-negative. FIV infection was defined as a positive PCR result from any sample or positive serology for anti-FIV Ab in an unvaccinated cat [30]. Progressive FeLV infection was defined as a positive result on p27 serology and detection of FeLV provirus on whole blood cPCR or qPCR recorded in the history. Regressive FeLV infection was defined as a negative result on p27 serology and a positive FeLV cPCR result for any tissue.

### 2.6. Age- and Sex-Matching

Control groups were matched to lymphoma cases for age and sex because being male and adult are both strong risk factors for FcaGHV1 detection [7,8,10]. Ages were tested for normality using the D’Agostino and Pearson Normality test. If both groups passed the normality test, then a *t*-test with Welch’s correction was used; if normality was not passed in either group, a Mann-Whitney U test was performed. A lottery blinded to all data except age and sex was used to exclude samples until age- and sex-matching was met (FFPE lymph node controls, *n* = 9; whole blood sample controls, *n* = 34). Sex-matching was considered successful when the male to female ratio of the control group was within 5 percentage points of the case group.

### 2.7. Statistical Analysis

FcaGHV1 infection status was compared between lymphoma cases (*n* = 122), and age- and sex-matched controls (*n* = 71), using Fisher’s exact test and the Odds Ratio (OR). To compare FcaGHV1 DNAemia between lymphoma blood samples (*n* = 93) and matched non-lymphoma controls (*n* = 52), Fisher’s exact test and OR were used. A Mann-Whitney U test was used to compare FcaGHV1 DNA loads. 

To investigate the distribution of FcaGHV1 DNA in lymphoma cases, 25 paired DNAs derived from FFPE lymphoma tissue and autologous uninvolved control tissue were available. McNemar’s exact test with OR was used for pairwise comparison of FcaGHV1 detection in lymphoma compared with autologous controls. The frequency of FcaGHV1 DNA detection in lymphoma tissues (*n* = 67) was also compared with that in matched control lymphoid tissues from cats without a cancer diagnosis (*n* = 19) using a Fisher’s exact test and OR. 

Survival analysis was performed for lymphoma cases that survived for greater than four days from diagnosis using a Kaplan-Meier estimate with a Log-rank (Mantel-Cox) test with death or euthanasia, or loss to follow up as end-points. Survival was compared between groups selected for the presence or absence FcaGHV1 DNAemia and the presence or absence of FIV-infection. The proportion of cases receiving multi-agent chemotherapy and other treatment categories combined (prednisolone alone, no treatment, or unknown) was calculated for each group, and Fisher’s exact test was used to compare treatments between groups in the survival analysis. Non-parametric Spearman’s correlation was used to compare the FcaGHV1 DNA load in whole blood to survival time.

For all cats in the study (cases and controls), a molecular diagnosis of FcaGHV1 infection was compared between indoor and outdoor cats, and between cats where fighting was observed or not observed using Fisher’s exact test and OR. All analyses were performed using GraphPad Prism (version 7.02 for Windows), and significance was determined at *p* = 0.05. 

## 3. Results

### 3.1. Viral Infection Status and Lymphoma Classification

**Figure 1:**
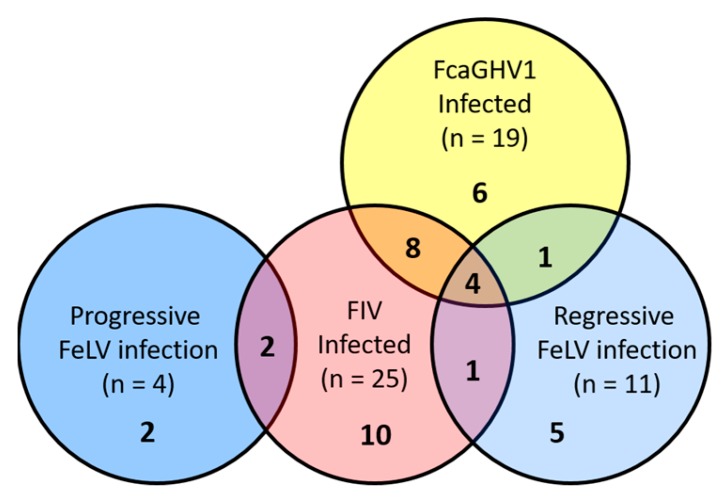
Infection status of lymphoma cases. The total number of infections by each virus is presented in parentheses. Single and coinfections are represented by the numbers in bold. Eighty-three cats with lymphoma tested negative for all 3 viruses.

**Figure 2:**
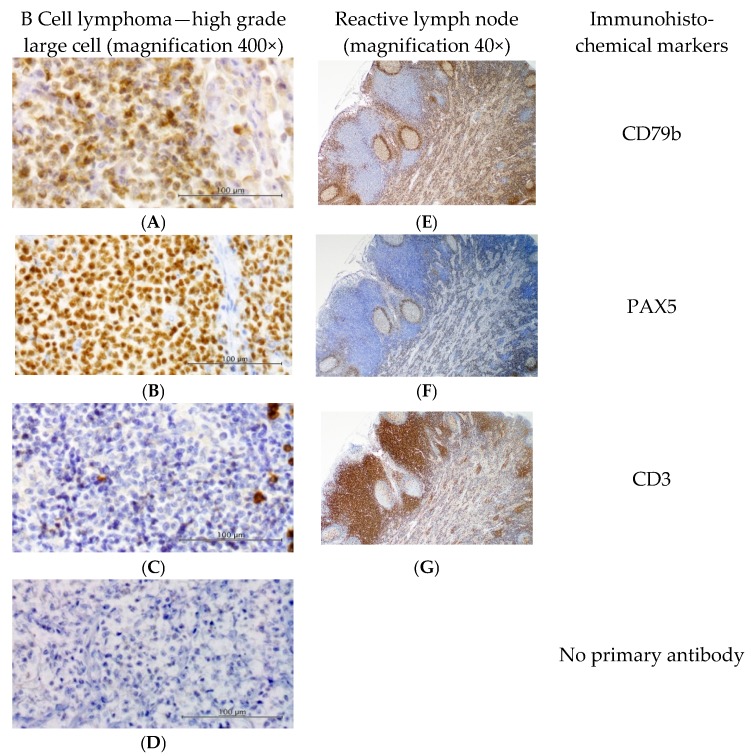
Immunohistochemical labelling. Results from a representative B cell lymphoma (left) demonstrate strong membranous labelling of proliferating cells with (**A**) anti-CD79b B-cell marker (1:5000), and nuclear labelling with (**B**) anti-PAX5 B-cell marker (1:100). (**C**) Anti-CD3 T-cell marker showed strong membranous staining of a few scattered small lymphocytes (1:400). Omission of the primary antibody (**D**) shows minimal background staining. The positive tissue control (**E**–**G**, reactive feline lymph node, right) demonstrates appropriate staining of B and T cells. Slides counterstained with Whitlock’s hematoxylin. Positive control sections shown at 40× magnification; all other images are 400× magnification.

The viral infection status of lymphoma cases is presented in Figure 1. Lymphoma cases diagnosed on histopathology comprised 68 high-grade, 10 intermediate-grade, and 12 low-grade; and the following immunophenotypes were identified: B-cell (*n* = 51; see Figure 2), T-cell rich B-cell (*n* = 3), T-cell (*n* = 9), epitheliotropic T-cell (*n* = 3), dual-staining (*n* = 1), null-cell (*n* = 2), and indeterminate (*n* = 5). 

### 3.2. Association between FcaGHV1 DNA Detection and Lymphoma 

**Figure 3:**
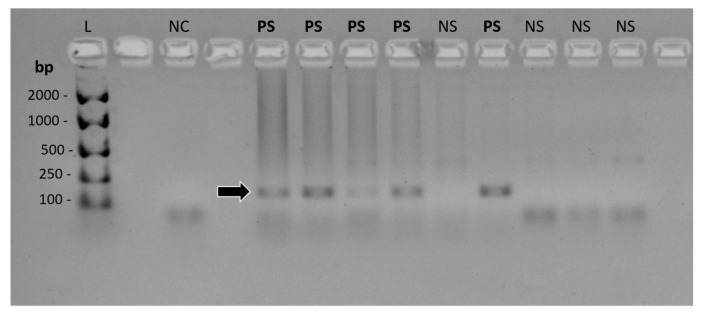
Conventional PCR for FcaGHV1. Agarose gel visualised under UV light, contrast inverted. The 164 bp product is identified by an arrow. Abbreviations: bp, base pair fragment length; L, DNA ladder-EasyLadder 1 (Bioline); NC, no-template (negative) control; PS, positive sample; NS, negative sample. Unmarked lanes are empty.

**Figure 4:**
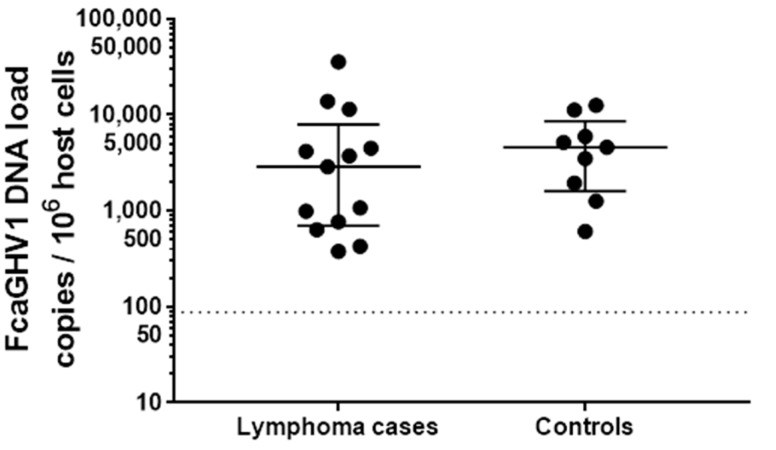
FcaGHV1 whole blood DNA loads in infected lymphoma cases (*n* = 13) and controls (*n* = 9) (*p* = 0.3933). Long bars represent the median and short bars the interquartile range for each group. The dotted line indicates the lowest viral load detected using this qPCR.

No difference was identified between the prevalence of FcaGHV1 DNA detection in cats which had a diagnosis of lymphoma compared with non-lymphomatous controls; a molecular diagnosis of FcaGHV1 was made in 19 of 122 cases (15.6%), compared with 10 of 71 controls matched for age and sex (14.1%) (*p* = 0.8373, OR = 1.125, 95% confidence interval [CI] = 0.4854 to 2.540). Representative FcaGHV1 cPCR results are shown in Figure 3. Using whole blood, 14 of 93 (15.1%) cats with lymphoma were FcaGHV1 DNAemic compared with 9 of 52 (17.3%) of matched controls (*p* = 0.8135, OR = 0.8467, 95%CI = 0.3491 to 2.008; see Table 1). Among FcaGHV1 DNAemic cats, there was no significant difference in FcaGHV1 DNA load between cats with or without lymphoma (*p* = 0.3933; Figure 4). 

The presence of FcaGHV1 DNA was distributed evenly between paired DNAs derived from lymphoma and autologous control tissues (*p* = 0.6831, OR = 1.000, 95%CI = 0.134 to 7.446; see Table 2). In fact, the number of cases in which the lymphoma was positive and control negative (*n* = 3), and vice-versa (*n* = 3), was the same. The remaining paired samples were negative in both tissues (*n* = 19). Similarly, the proportion of lymphoma DNAs positive for FcaGHV1 (10/67, 14.9%) was comparable with that in non-neoplastic lymph node DNAs (2/19, 10.5%) (*p* > 0.9999, OR = 1.491, 95%CI = 0.3331 to 7.309). 

### 3.3. FeLV Infection

FeLV provirus was detected by cPCR in 13 of 42 (31.0%) lymphoma cases where at least one FFPE tissue sample was available for testing, and point-of-care FeLV antigen test results from whole blood were also available. Of these, 11 (26.2%) were diagnosed with regressive FeLV infection and two cases (4.8%) were progressively infected. Two of the lymphoma cases with only blood samples available were also identified as having progressive FeLV infection.

### 3.4. FcaGHV1 DNAemia and Survival

**Figure 5:**
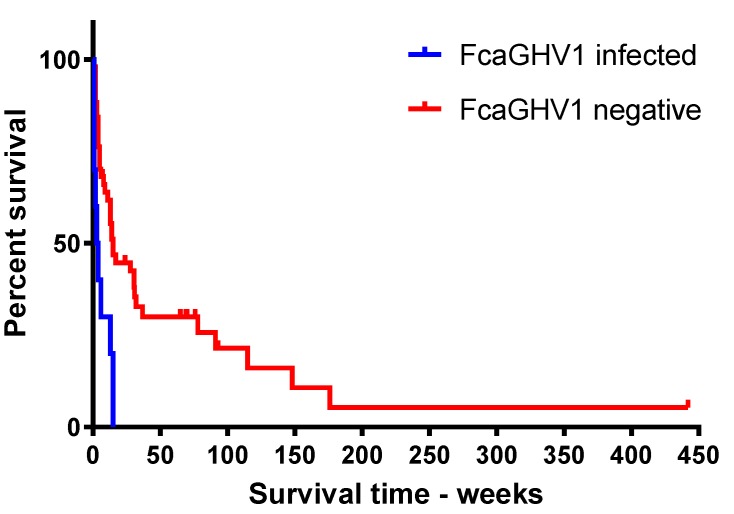
Kaplan-Meier survival analysis of lymphoma cases surviving longer than four days—FcaGHV1 DNAemic (*n* = 10) vs. FcaGHV1 negative (*n* = 51; *p* = 0.0019).

Among lymphoma cases, a finding of FcaGHV1 DNAemia (*n* = 10; median survival time (MST) = 3.5 weeks, range = 1 to 15 weeks) was associated with significantly shorter survival compared with FcaGHV1 qPCR negative cases (*n* = 51; *p* = 0.0019; MST = 14 weeks, range = 1 to 442 weeks; Figure 5). To investigate whether the observed difference in survival might be attributable to differences in treatment received, treatment categories for FcaGHV1 DNAemic versus qPCR negative groups were compared (Table 3). No significant difference in treatment was detected between FcaGHV1 DNAemic and the FcaGHV1 negative groups (*p* = 0.3669). 

Of the 10 FcaGHV1 DNAemic lymphoma cases in the survival analysis, four were coinfected with FIV and one with regressive FeLV and two with both FIV and regressive FeLV. In comparison, amongst the 51 FcaGHV1 negative lymphoma cases in the survival analysis, seven cases were FIV infected, one case was progressively FeLV infected, one with FIV and progressive FeLV and one case with regressive FeLV infection. The small numbers of cases in each subgroup precluded meaningful analysis of these co-infection status. However, when the effect of FIV alone on survival time was analysed, there was no significant difference between FIV-infected and FIV-uninfected cases (*p* = 0.0609). In addition, progressive FeLV infection, which carries a poor prognosis, was not identified in FcaGHV1 DNAemic lymphoma cases, so reduced survival in this group could not be attributed to this cause.

### 3.5. Outdoor Access and Fighting History as Risk Factors for FcaGHV1 Detection 

FcaGHV1 DNA detection was significantly higher in cats with a known history of fighting (*n* = 29) compared with those which had no history of fighting (*n* = 208; *p* = 0.0443, OR = 2.667, 95%CI = 1.092 to 6.448). Similarly, a molecular diagnosis of FcaGHV1 was significantly more likely in cats with possible or known outdoor access (15.0%, 31/207) compared with those kept strictly indoors (0.0%, 0/30; *p* = 0.0182, OR = infinity, 95%CI = 1.453 to infinity).

## 4. Discussion

This is the first study to investigate FcaGHV1 detection in cats diagnosed with lymphoma. Neither detection of FcaGHV1 DNA nor whole blood virus load was related to a diagnosis of lymphoma in this study. While the results of disease association studies like this one can support causality, the absence of an association does not rule out a pathogenic role for a virus, particularly when the virus has a high prevalence in the general population [31]. For example, the seroprevalence of EBV, a group 1 carcinogen that is causal in a range of lymphomas and nasopharyngeal carcinoma, is greater than 90% in adults, yet EBV-associated neoplasia is an extremely rare outcome of infection, so detection of EBV infection is unrelated to cancer risk [32,33]. Instead, an aetiological role for EBV can be can confirmed using in situ hybridization to identify latency-associated transcripts in tumour cells. In addition, measurement of EBV load in peripheral blood mononuclear cells, plasma or whole blood has diagnostic and prognostic utility in some EBV-associated lymphomas [34,35,36,37].

The true prevalence of FcaGHV1 remains to be determined, but studies to date suggest that infection is widespread in domestic cat populations. The molecular prevalence of FcaGHV1 in whole blood using qPCR targeting the *glycoprotein B* gene in cats from Australia, Singapore, USA, central Europe and UK, ranges from 9.6–16.2% [7,8,9], and the seroprevalence is estimated to be around twice that of FcaGHV1 DNAemia [38]. Any association between FcaGHV1 and lymphoma might, therefore, be more readily observed by detecting viral DNA and virus load rather than using serology. The molecular prevalence of FcaGHV1 in both lymphoma cases and controls was similar to that reported previously in cats which were not selected for disease presentation. These results do not exclude FcaGHV1 involvement in specific subtypes of feline lymphoma. Consensus on the sub-classification of clinically aggressive lymphomas in cats is emerging slowly in comparison to that in the diagnosis of human lymphoproliferative diseases [24,39,40,41]. The criteria used here to recruit lymphoma cases selected for clinically aggressive tumours and excluded lymphocytic low-grade gastrointestinal lymphoma (LGAL), an increasing common diagnosis in cats over the past 20 years [42]. LGAL typically has an indolent course and a good to excellent prognosis with treatment [43]. Most lymphomas were of the B-cell immunophenotype, which is consistent with other reports since the control of FeLV infection [19,44,45].

We found no evidence that FcaGHV1 DNA is distributed preferentially in lymphoma tissue. Early evidence of an oncogenic role for KSHV was afforded by the identification of gammaherpesvirus DNA in Kaposi’s sarcoma tissue but was not uninvolved autologous tissue [46]. Unlike KSHV infection, which is limited to specific patient subgroups, FcaGHV1 seems to be a common infection, and our data support results from a previous study suggesting that viral DNA is found in many tissues in infected cats [7].

The observation that FcaGHV1 DNAemia was associated with significantly reduced long-term survival in lymphoma is intriguing. Investigation of this relationship in a larger, prospective study would assist in understanding whether FcaGHV1 DNAemia could have clinical utility as a negative prognostic marker in feline lymphoma, regardless of the nature of any association. Similar findings have been reported for EBV where DNAemia was associated with reduced survival in patients with diffuse large B-cell lymphoma, regardless of whether EBV DNA was detected in the tumour [47]. FIV infection is a strong risk factor for FcaGHV1 DNAemia, so the frequent documentation of dual infections, including six of 10 lymphoma cases in the survival analysis, was expected [7,8,25]. Co-infections were more common in the FcaGHV1-infected lymphoma cases than in the FcaGHV1 negative cases, which could contribute to poorer outcomes through an unknown mechanism. Conclusions regarding the possible contribution of each virus to the observed reduced survival are precluded by the small numbers of co-infected cases in this analysis, although survival in lymphoma cases infected with FIV infection alone was not significantly reduced. Similarly, FIV-infection was not associated with reduced survival in feline lymphoma cases treated with multiagent chemotherapy in a previous study, although FcaGHV1 status was not investigated as the study was conducted prior to the discovery of FcaGHV1 [41].

Our finding that four of 122 lymphoma cases had progressive FeLV infection is consistent with that reported previously in cats with lymphoma in the region and is in line with the prevalence of the virus in the region [48,49]. Progressive FeLV infection is unequivocally lymphomagenic and infection carries a guarded prognosis regardless of whether lymphoma is diagnosed [50,51,52,53]. Eleven out of the 42 lymphoma cases with both FFPE tissue samples and FeLV antigen blood test results were diagnosed with regressive FeLV infection. This is higher than the background prevalence which has been recorded previously in Australia (<3%), as well as in Germany (<3%), Switzerland and the UK (up to 10%) [54,55,56,57,58,59]. Regressive FeLV infection, where provirus is contained but not completely eliminated, likely reflects a strong host immune response following exposure [60]. Potential exists for regressive infection to contribute to lymphomagenesis because provirus integration and low-level replication occur. Evidence from previous studies is conflicting, with some demonstrating high detection rates for FeLV provirus in lymphoma arising in seronegative cats and others not [44,48,52,54,61,62,63]. This includes a previous study conducted by our institution where regressive FeLV infection was identified in 19 of 86 (22%) feline lymphomas [48]. Our results support further investigation of a role for regressive FeLV infection in tumorigenesis in cats.

This study provided additional epidemiological data for FcaGHV1 by identifying opportunities for fighting and roaming as risk factors for FcaGHV1 detection. Transmission of FcaGHV1 during aggressive encounters has been suggested previously because male sex and coinfection with FIV or haemotropic mycoplasmas are risk factors for both FcaGHV1 DNAemia and fighting [7,8,9,64]. These findings lend support to aggressive contact as a possible route of FcaGHV1 transmission. Longitudinal studies of both FcaGHV1 DNAemic and FcaGHV1-negative cats may allow the identification of additional risk factors for infection, as well as further investigation of associations with disease.

The size and retrospective nature of this study should be taken into account when interpreting the findings. Some analyses involve groups with a small number of cases. Nonetheless, the data provide a snapshot of the possible influences of FcaGHV1 in spontaneous disease in relation to established viral risk factors for lymphoma. Studies using well-curated subsets of lymphoma from different regions and identification of the cellular location and transcriptional activity of FcaGHV1 within tumour tissue are supported.

## Figures and Tables

**Table 1:** FcaGHV1 positive lymphoma cases and controls by sample type.

	Sample Type	Total	FcaGHV1 Positive (%)
**Lymphoma cases**	FFPE lymphoma tissue	67	10 (14.9)
	Blood	93	14 (15.1)
**Controls**	FFPE lymph node	19	2 (10.5)
	Blood	52	9 (17.3)

FFPE = formalin-fixed, paraffin embedded.

**Table 2:** Paired-sample combinations analysed for FcaGHV1 DNA distribution in lymphoma compared to autologous non-lymphoma tissue using McNemar’s exact test.

Lymphoma Tissue FcaGHV1 Positive	Autologous Tissue FcaGHV1 Positive	Number of Paired Samples
Yes	Yes	0
Yes	No	3
No	Yes	3
No	No	19

**Table 3:** Treatment status of FcaGHV1 positive and negative lymphoma cases with survival and treatment data available.

Treatment Group	FcaGHV1 DNAemic *n* = 10 (%)	FcaGHV1 Negative *n* = 51 (%)
Multiagent chemotherapy	7 (70.0%)	43 (84.3%)
Prednisolone	1 (10.0%)	3 (5.9%)
No treatment	1 (10.0%)	3 (5.9%)
Unknown	1 (10.0%)	2 (3.9%)
